# Revisiting the Sarcopenic Index in Older Adults with Reduced Kidney Function: Association with EWGSOP2-Defined Probable Sarcopenia

**DOI:** 10.3390/jcm15051782

**Published:** 2026-02-26

**Authors:** Diana Moldovan, Ina Kacso, Cosmina Bondor, Lucreția Avram, Dana Crişan, Ariana Condor, Crina Rusu, Alina Potra, Dacian Tirinescu, Maria Ticala, Yuriy Maslyennikov, Valer Donca

**Affiliations:** 1 Department of Nephrology, Faculty of Medicine, Iuliu Hatieganu University of Medicine and Pharmacy, 400012 Cluj-Napoca, Romaniaalina.potra@umfcluj.ro (A.P.); cosa.maria@umfcluj.ro (M.T.);; 2 Emergency County Hospital, 400034 Cluj-Napoca, Romania; 3 Department of Medical Informatics and Biostatistics, Iuliu Hatieganu University of Medicine and Pharmacy, 6 Pasteur Street, 400349 Cluj-Napoca, Romania; cbondor@umfcluj.ro; 4 Department of Geriatrics—Gerontology, Faculty of Medicine, Iuliu Hatieganu University of Medicine and Pharmacy, 400012 Cluj-Napoca, Romania; valer.donca@umfcluj.ro; 5 Clinical Municipal Hospital, 400139 Cluj-Napoca, Romania; 6 Department of Internal Medicine, 5th Medical Clinic, Faculty of Medicine, Iuliu Hatieganu University of Medicine and Pharmacy, 400012 Cluj-Napoca, Romania

**Keywords:** sarcopenic index, probable sarcopenia, EWGSOP2, older adults, renal dysfunction

## Abstract

**Background:** Sarcopenia is highly prevalent in older adults and in individuals with impaired kidney function, where it is associated with adverse clinical outcomes. A creatinine–cystatin C–based sarcopenic index has been proposed as a surrogate marker of muscle status; however, its association with sarcopenia as defined by the EWGSOP2 framework, particularly in the context of renal dysfunction, remains uncertain. **Methods:** Older adults were classified according to EWGSOP2 criteria into probable, confirmed, and severe sarcopenia. Associations between the sarcopenic index and sarcopenia phenotypes were examined using group comparisons and multivariable logistic regression analyses in the overall cohort and in a subgroup of participants with an estimated glomerular filtration rate (eGFR) < 60 mL/min/1.73 m^2^. **Results:** The sarcopenic index was not independently associated with probable, confirmed, or severe sarcopenia. In contrast, age emerged as the strongest independent correlate of probable sarcopenia (OR 1.12; 95% CI 1.05–1.19, *p* = 0.001), while body mass index was independently associated with confirmed sarcopenia (OR 0.91; 95% CI 0.86–0.96, *p* < 0.001). Similar patterns were observed in participants with reduced kidney function. **Conclusions:** Within the present analytical framework, the sarcopenic index did not show a meaningful association with EWGSOP2-defined probable sarcopenia, the most uniformly assessable EWGSOP2 stage, in older adults, including those with reduced kidney function. Exploratory analyses of more advanced sarcopenia stages did not reveal additional associative information. These findings should be interpreted within a descriptive and associative framework rather than a formal assessment of diagnostic or clinical decision-making performance.

## 1. Introduction

Sarcopenia is a progressive skeletal muscle disorder characterized by reduced muscle strength, low muscle quantity and quality, and impaired physical performance, and is strongly associated with disability, hospitalization, and mortality in older adults [1,2]. The adoption of consensus definitions has improved clinical recognition; however, sarcopenia remains underdiagnosed in routine practice, partly due to the need for structured assessments and the heterogeneity of available screening tools [3]. In addition to the EWGSOP2 framework, other regional definitions such as the 2019 update from the Asian Working Group for Sarcopenia (AWGS) emphasize the relevance of the phenotypic classification and the importance of pragmatic diagnostic pathways [4].

Chronic kidney disease (CKD) represents a high-risk clinical condition for the development of sarcopenia. Muscle wasting and dysfunction are frequent in CKD and are driven by inflammation, metabolic complications, hormonal dysregulation, reduced physical activity, and nutritional deficits [5,6]. The epidemiology of sarcopenia in CKD has been documented across the continuum from non-dialysis CKD to dialysis, with prevalence estimates varying substantially according to diagnostic criteria and assessment modality [7]. The evidence from meta-analysis confirms a high burden among dialysis patients and supports the association between sarcopenia and cardiovascular mortality and events [8,9]. Importantly, in non-dialysis CKD, sarcopenia is associated with increased risk of end-stage kidney disease and death [10], while systematic reviews in all CKD stages link sarcopenia to adverse clinical outcomes [11,12]. Additionally, the coexistence of CKD and sarcopenia appears to synergistically increase cardiovascular risk and mortality [13].

Given this burden, clinically applicable screening strategies are essential. The EWGSOP2 algorithm proposes a stepwise model distinguishing probable sarcopenia (low strength), confirmed sarcopenia (low strength plus low muscle mass), and severe sarcopenia (confirmed sarcopenia plus poor physical performance) [1]. However, implementing these criteria in clinical nephrology remains challenging. While muscle strength testing such as handgrip strength and chair stand tests is feasible, muscle mass evaluation often requires equipment and expertise not universally available. This has stimulated interest in simplified screening instruments such as SARC-F or its variants, as well as alternative bedside tests, including the Ishii score [14,15,16,17]. However, screening tools are designed to prioritize sensitivity and may require confirmatory tests, reinforcing the need for reliable and scalable biomarkers or surrogate indices.

Body composition assessment modalities also differ in terms of feasibility and accuracy. Bioelectrical impedance analysis (BIA) is practical for estimating appendicular skeletal muscle mass (ASM) and ASM index (ASMI), but its performance may be influenced by hydration status—an important issue in CKD [5]. Imaging-based approaches offer complementary information: abdominal computed tomography (CT)–derived skeletal muscle measurements have been associated with clinically relevant outcomes and can be leveraged opportunistically when scans are available [18,19]. These developments illustrate the active search for scalable approaches that balance diagnostic precision and clinical workload.

A parallel line of research has proposed biochemical surrogate indices for muscle status. The creatinine–cystatin C-based sarcopenic index is conceptually attractive because serum creatinine is partly determined by muscle mass, whereas cystatin C is considered less dependent on muscle mass and more reflective of glomerular filtration [20]. Early studies reported associations between the sarcopenic index and mortality in critically ill patients [20,21], and subsequent cohorts linked the index to cardiovascular and all-cause mortality or stroke risk [22,23]. Importantly, the sarcopenic index has also been studied as a predictor of incident CKD in community populations, suggesting complex bidirectional relationships between muscle status, biomarkers, and renal outcomes [24]. In addition, related constructs such as the creatinine/cystatin C ratio have been explored in other clinical contexts, such as oncology, as screening and prognostic tools, underscoring broad interest in this biomarker approach [25].

Nutrition and lifestyle factors further complicate sarcopenia assessment in CKD. Nutritional restrictions, anorexia, and protein-energy wasting contribute to muscle loss, while clinicians shape dietary management and adherence [26]. These intervention-focused data reinforce the clinical importance of identifying sarcopenia early and accurately, especially in patients with eGFR < 60 mL/min/1.73 m^2^, where risk and potential benefit of intervention are substantial.

Therefore, the primary aim of this study was to examine the association between a creatinine–cystatin C–based sarcopenic index and EWGSOP2-defined probable sarcopenia in a large cohort of older adults. Secondary exploratory objectives were to assess its associations with confirmed and severe sarcopenia in subgroups with available data and in participants with reduced kidney function.

## 2. Materials and Methods

### 2.1. Study Design and Population

This was a cross-sectional observational study conducted in older adults evaluated in a geriatric clinical setting. Consecutive patients aged ≥65 years with available functional, anthropometric, imaging, and biochemical assessments were eligible for inclusion. Patients with acute illness, acute kidney injury, active infection, malignancy under active treatment, dialysis dependency, intensive care admission, or incomplete sarcopenia assessment were excluded. The final analytical cohort included 396 participants. For subgroup analyses, patients were stratified according to kidney function, with particular focus on those with estimated glomerular filtration rate (eGFR) < 60 mL/min/1.73 m^2^. Diabetes mellitus was defined based on documented medical history at the time of assessment.

Sarcopenia was defined and classified according to the European Working Group on Sarcopenia in Older People 2 (EWGSOP2) consensus criteria. Probable sarcopenia was defined by low muscle strength. Confirmed sarcopenia was defined by low muscle strength combined with low muscle mass. Severe sarcopenia was defined by confirmed sarcopenia and impaired physical performance.

Muscle strength was assessed using handgrip strength (HGS), measured with a calibrated handheld dynamometer (model MVS-12-0241) in the dominant hand, using standardized testing procedures. Measurements were performed with the participant in a seated position, shoulder adducted, elbow flexed at 90°, and wrist in a neutral position. Participants were instructed to squeeze the dynamometer with maximal effort. Three measurements were obtained for each hand, with the highest value retained for analysis. Low muscle strength was defined according to EWGSOP2 cut-off values (<27 kg for men and <16 kg for women). Lower limb muscle strength was assessed using the five-times chair stand test. Participants were asked to rise from a seated position and sit down five times as quickly as possible, with arms crossed over the chest. The time required to complete the test was recorded. Poor performance was defined as a chair stand time > 15 s, in accordance with EWGSOP2 recommendations. Given the geriatric clinical setting, a high prevalence of impaired muscle strength was anticipated and reflects the characteristics of a referred older adult population rather than community-dwelling individuals.

Muscle mass was assessed using bioelectrical impedance analysis (BIA), performed under standardized conditions. We used a validated device, the Visbody S30 Bioelectrical Impedance Body Composition Analyser Scanner (Xi’an, China). Measurements were obtained with participants in the supine position after a resting period. Appendicular skeletal muscle mass (ASM) was calculated using validated equations, and the appendicular skeletal muscle mass index (ASMI) was derived by dividing ASM by height squared (kg/m^2^). Low muscle mass was defined using EWGSOP2 cut-off values (<7.0 kg/m^2^ for men and <5.5 kg/m^2^ for women). In a subset of patients with available imaging, computed tomography (CT) scans were used to assess muscle mass at the level of the abdominal wall from a single slice at the lumbar L3 level. Siemens Healthineers (Forchheim, Germany). SOMATOM go Up was the CT scanner used for this evaluation. Cross-sectional images were analyzed to quantify muscle area using standard attenuation thresholds for skeletal muscle. Using abdominal CT scan images, skeletal muscle area (SMA, cm ^2^) was used to quantify the skeletal muscle mass. Personalized indexes for SMA, were normalized by height squared to calculate the skeletal muscle index (SMI, cm^2^/m^2^). CT-derived muscle measurements were used as complementary information for muscle mass assessment. Physical performance was assessed using the Short Physical Performance Battery (SPPB), which includes tests of balance, gait speed, and chair stands. Each component was scored from 0 to 4, yielding a total score ranging from 0 to 12. Poor physical performance was defined as an SPPB score ≤ 8, in line with EWGSOP2 criteria, and was used to classify severe sarcopenia.

Although the EWGSOP2 framework was used as the conceptual reference, only probable sarcopenia, based on muscle strength assessment, could be consistently evaluated in the entire cohort. Assessments required for confirmed and severe sarcopenia (muscle mass and physical performance) were available only in subsets of participants. Accordingly, analyses of confirmed and severe sarcopenia were considered secondary and exploratory.

The sarcopenic index (SI) was calculated as the ratio of serum creatinine to serum cystatin C. Serum creatinine was measured in mg/dL and cystatin C in mg/L. The sarcopenic index was calculated as the ratio of serum creatinine to serum cystatin C, multiplied by 100, without unit conversion. Given the influence of kidney function on creatinine levels, higher values do not necessarily reflect greater muscle mass in individuals with reduced eGFR. Accordingly, the sarcopenic index was analyzed as a continuous variable without assuming specific diagnostic cut-offs.

Clinical and biochemical measurements. Baseline data included age, sex, body mass index (BMI, kg/m^2^), and routine laboratory parameters, including urinary albumin over creatinine ration (UACR) and lipid profile parameters (total cholesterol, LDL-cholesterol, HDL-cholesterol, and triglycerides). Kidney dysfunction was characterized using serum urea, creatinine, cystatin C, and uric acid. The estimated glomerular filtration rate (eGFR) was obtained from the CKD-Epidemiology Collaboration (EPI) 2021 equation for creatinine and one for creatinine–cystatin C. eGFR was considered as a continuous and categorical variable. Reduced kidney function was defined based on a single estimated glomerular filtration rate (eGFR) measurement < 60 mL/min/1.73 m^2^, acknowledging that chronicity could not be formally established. For subgroup analyses, patients were stratified into eGFR categories: <60 mL/min/1.73 m^2^, <45 mL/min/1.73 m^2^ and <30 mL/min/1.73 m^2^.

### 2.2. Statistical Analysis

Statistical analyses were performed using IBM SPSS Statistics, version 25.0. Continuous variables were tested for normality using the Shapiro–Wilk test and are presented as mean ± SD or median (IQR), as appropriate. Qualitative variables are reported as frequencies and percentages. Group comparisons were conducted using Student’s *t*-test or Mann–Whitney *U* test for continuous variables and χ^2^ or Fisher’s exact test for qualitative variables.

Associations between the sarcopenic index and sarcopenia phenotypes were evaluated. To identify independent predictors and assess the validity of the index, multivariable logistic regression models were constructed, adjusting for age, sex, BMI, nutritional and inflammatory markers, and functional parameters. Multivariable logistic regression models (Enter method) were constructed for probable, confirmed, and severe sarcopenia. Results are presented as odds ratios (ORs) with 95% confidence intervals (CIs). Subgroup analyses were conducted in patients with eGFR < 60 mL/min/1.73 m^2^, with exploratory analyses in those with eGFR < 45 and <30 mL/min/1.73 m^2^. Multicollinearity among predictors was assessed using variance inflation factors (VIFs) computed from an equivalent linear regression models including the same covariates; VIFs values < 2 were considered acceptable. All statistical tests were two-sided, and *p* < 0.05 was considered statistically significant.

### 2.3. Ethics

The study was conducted in accordance with the Declaration of Helsinki and was approved by the Ethics Committee of the Municipal Clinical Hospital Cluj-Napoca (protocol no. 29/2020; date of approval: 16 October 2020).

## 3. Results

### 3.1. Prevalence of Sarcopenia According to EWGSOP2 Criteria

Results are presented with probable sarcopenia as the primary outcome, while findings related to confirmed and severe sarcopenia reflect exploratory analyses performed in subgroups with available data.

Among the total cohort of 396 participants, probable sarcopenia was identified in 347 individuals (87.6%), while 49 participants (12.4%) did not meet the criteria. Reduced muscle strength was the most frequent abnormality, with low handgrip strength (HGS < 27 kg in men and <16 kg in women) observed in 262 of 396 evaluated subjects (66.1%), and prolonged five-times chair stand test (>15 s) in 222 of 314 participants (70.7%).

Confirmed sarcopenia was diagnosed in 95 of 218 participants (43.6%). Regarding muscle quantity and quality, reduced appendicular skeletal muscle mass (ASM < 20 kg in men and <15 kg in women) was present in 12 of 142 participants (8.5%), while low appendicular skeletal muscle index (ASMI < 7.0 kg/m^2^ in men and <5.5 kg/m^2^ in women) was identified in 48 subjects (33.8%). A CT-derived low muscle mass was observed in 86 of 157 participants (54.8%).

Severe sarcopenia, defined by impaired physical performance, was present in 25 of 178 subjects (14%). Low gait speed (≤0.8 m/s) was detected in 251 of 284 participants (88.4%), SPPB score ≤ 8 in 340 of 396 subjects (85.8%), and prolonged Timed Up and Go (TUG ≥ 20 s) in 202 of 374 participants (54.0%) (Table 1).

### 3.2. Correlation Between Sarcopenic Index and Sarcopenia Criteria

**Figure 1:**
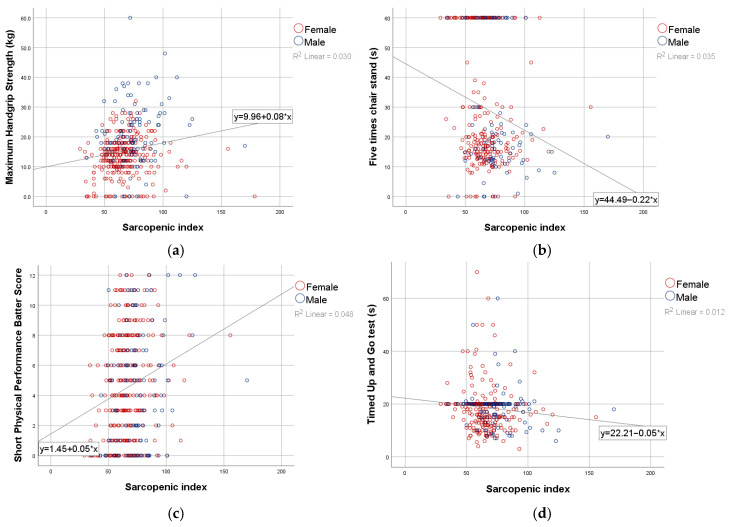
Monotonic association between sarcopenic index and functional parameters: HGS (**a**), Five times chair stand (**b**), SPPB Score (**c**) and TUG (**d**).

In the total cohort, the sarcopenic index showed significant correlations with muscle strength and physical performance parameters with the exception of gait speed, but not with muscle mass indices (Table 2). Specifically, the sarcopenic index correlated weakly and positively with handgrip strength (Spearman’s r = 0.21, *p* < 0.001) and SPPB score (r = 0.21, *p* < 0.001), and weakly and negatively with the five-times chair stand test (r = −0.17, *p* = 0.002) and TUG (r = −0.13, *p* = 0.019). Significant monotonic associations were observed (Figure 1).

No statistically significant correlations were found between the sarcopenic index and muscle quantity or quality parameters, including ASM, ASMI, and CT-derived muscle mass.

In sex-stratified analyses, these associations remained significant only in women, whereas no significant correlations were observed in men (Table 2).

### 3.3. Clinical and Biochemical Characteristics in Probable Sarcopenia

Participants with probable sarcopenia were significantly older, had lower body mass index (BMI), hemoglobin, albumin, vitamin D and worse kidney function (lower eGFR and higher cystatin C) and higher inflammatory markers (CRP), compared with those without probable sarcopenia (Table 3).

Lipid profile parameters (total cholesterol, LDL-cholesterol, HDL-cholesterol, and triglycerides), uric acid and sarcopenic index did not differ significantly between participants with and without probable sarcopenia. The prevalence of diabetes mellitus did not differ significantly according to probable sarcopenia status.

In multivariable logistic regression analysis for probable sarcopenia, age was independently associated with probable sarcopenia (OR 1.12, 95% CI 1.05–1.19, *p* = 0.001). No independent associations were observed for the sarcopenic index, body mass index, estimated GFR based on creatinine, cystatin C, hemoglobin, albumin, vitamin D or C-reactive protein (Supplementary Table S1).

### 3.4. Patients with eGFR < 60 mL/min/1.73 m^2^

In patients with an estimated GFR below 60 mL/min/1.73 m^2^, those with probable sarcopenia were significantly older, had lower serum albumin levels concentrations and higher UACR, and lower CysCr-based eGFR compared with those without probable sarcopenia. No significant differences were observed for sex distribution, body mass index, hemoglobin, lipid profile, higher CRP, creatinine-based eGFR, or sarcopenic index (Table 4).

In multivariable logistic regression analysis performed in patients with eGFR < 60 mL/min/1.73 m^2^, age remained independently associated with probable sarcopenia (OR 1.10, 95% CI 1.01–1.21, *p* = 0.037). No independent associations were identified for the sarcopenic index, albumin, UACR or cystatin C–based eGFR. Given the small number of participants without probable sarcopenia in the reduced kidney function subgroup, multivariable analyses should be interpreted cautiously (Supplementary Table S2).

### 3.5. Confirmed Sarcopenia

Patients with confirmed sarcopenia were significantly older and had lower BMI and albumin levels, and higher cystatin C levels compared with those without confirmed sarcopenia. No significant differences were observed for sex distribution, renal function categories, inflammatory markers, or sarcopenic index (Table 5).

In multivariable logistic regression analysis, BMI was independently associated with confirmed sarcopenia (OR 0.905, 95% CI 0.856–0.957, *p* < 0.001). No independent associations were observed for age, albumin, cystatin C, eGFR (CysCr) or the sarcopenic index (Supplementary Table S3).

### 3.6. Patients with Confirmed Sarcopenia and eGFR < 60 mL/min/1.73 m^2^

Among patients with reduced kidney function, confirmed sarcopenia was associated with older age, lower BMI, higher uric acid and vitamin D concentrations (Table 6).

Multivariable logistic regression. In patients with eGFR < 60 mL/min/1.73 m^2^, BMI remained independently associated with confirmed sarcopenia (OR 0.879, 95% CI 0.794–0.972, *p* = 0.012). No independent associations were observed for age, uric acid, vitamin D, or the sarcopenic index.

### 3.7. Severe Sarcopenia

Severe sarcopenia was associated with advanced age, lower BMI and albumin levels, reduced renal function, and higher cystatin C levels. No significant differences were observed for sex distribution or sarcopenic index (Table 7).

In multivariable analysis, age was independently associated with severe sarcopenia (OR 1.12, 95% CI 1.001–1.248, *p* = 0.048). No independent associations were identified for BMI, albumin, renal function, cystatin C, or the sarcopenic index (Supplementary Table S4).

### 3.8. Patients with Severe Sarcopenia and eGFR < 60 mL/min/1.73 m^2^

In patients with eGFR < 60 mL/min/1.73 m^2^, severe sarcopenia was associated with older age, and showed a trend toward lower sarcopenic index values (*p* = 0.054) (Table 8).

In multivariable analysis restricted to patients with reduced kidney function, no variable was statistically significant. The sarcopenic index was not independently associated.

For subgroup categories with eGFR < 45 mL/min/1.73 m^2^ and <30 mL/min/1.73 m^2^, sarcopenic index was not predictive for any sarcopenia phenotypes or features.

## 4. Discussion

In this large cohort of older adults, we evaluated the association between a creatinine–cystatin C–based sarcopenic index and probable sarcopenia-the only EWGSOP2 stage that could be robustly and consistently assessed in the entire population. The principal finding was the lack of an independent association between the sarcopenic index and probable sarcopenia, both in the overall cohort and in participants with reduced kidney function. Analyses of confirmed and severe sarcopenia, performed as exploratory evaluations in subgroups, yielded consistent negative results and should be interpreted in light of data availability constraints.

### 4.1. The Sarcopenic Index and EWGSOP2-Defined Sarcopenia: Limited Associative Evidence

The sarcopenic index was proposed as a pragmatic surrogate of muscle mass, based on the assumption that serum creatinine reflects muscle production, while cystatin C is largely independent of muscle mass and more accurately reflects glomerular filtration [20]. Initial studies demonstrated associations between lower sarcopenic index values and adverse outcomes, including mortality, in critically ill or hospitalized patients [20,21]. Subsequent population-based analyses extended these observations to cardiovascular risk [22]. From a nephrological perspective, this construct is intuitively appealing, as both biomarkers are routinely measured in kidney diseases and are central to renal risk stratification.

However, our data indicate that this theoretical advantage does not translate into ability to provide meaningful association with EWGSOP2-defined sarcopenia. In our study, the sarcopenic index did not differ between sarcopenic and non-sarcopenic individuals and showed no significant associations with confirmed or severe sarcopenia. These findings are consistent with earlier reports demonstrating that the sarcopenic index cannot accurately detect low muscle mass or sarcopenia defined by consensus criteria in community-dwelling older adults [27]. Similarly, data from the UK Biobank revealed statistically significant but clinically weak diagnostic performance [28].

Importantly, in kidney disease the determinants of both creatinine and cystatin C extend far beyond muscle mass. Creatinine is influenced by dietary protein intake, tubular secretion, metabolic acidosis, and reduced physical activity, while cystatin C is strongly modulated by inflammation, adiposity, thyroid dysfunction, and corticosteroid exposure [5]. In CKD, the confounders such as inflammation, oxidative stress, insulin resistance, and hormonal disturbances are amplified, further reducing the specificity of creatinine–cystatin C–based indices for muscle assessment [6,29]. As sarcopenia progresses, low muscle quality, neuromuscular dysfunction, and impaired physical performance become dominant features—dimensions that are not captured by a static biochemical ratio. Thus, in CKD, the sarcopenic index is more likely to reflect overall disease burden rather than true skeletal muscle status.

### 4.2. Correlation with Function but Not Muscle Mass

In the present study, the sarcopenic index showed weak correlations with muscle strength and physical performance measures, including handgrip strength, chair stand test, SPPB score, and TUG, but no association with direct measures of muscle quantity or quality (ASM, ASMI, or CT-derived indices). These correlations were modest in magnitude and largely confined to women.

This dissociation supports the notion that the sarcopenic index may reflect physiological reserve or illness severity rather than true skeletal muscle mass. Similar discrepancies have been reported in geriatric cohorts, where related ratios (creatinine/cystatin C) were more strongly associated with functional decline than with imaging-based muscle measures [30]. Nevertheless, evidence regarding its relevance remains mixed, and some studies suggest that related ratios may outperform the sarcopenic index in geriatric assessments, particularly in the setting of sarcopenic obesity [30]. Importantly, such associations do not meet the requirements of a diagnostic biomarker for sarcopenia as defined by EWGSOP2 [1]. This dissociation is particularly relevant in CKD, where functional decline may precede or progress independently of measurable muscle mass loss.

### 4.3. Sarcopenia Phenotypes and the Role of Kidney Dysfunction

Sarcopenia was highly prevalent in our cohort, with a predominance of probable sarcopenia driven by impaired muscle strength. At this early stage, age emerged as the primary determinant, while BMI, biochemical markers, and the sarcopenic index showed no independent associations. This pattern aligns with the EWGSOP2 framework, which emphasizes that early sarcopenia is largely driven by neuromuscular dysfunction preceding overt muscle mass loss [1,2].

In confirmed sarcopenia, BMI was the only variable independently associated with the diagnosis, both in the overall cohort and in patients with eGFR < 60 mL/min/1.73 m^2^. Notably, renal function estimates and the sarcopenic index did not retain independent associations. These findings are particularly relevant because sarcopenia in non-dialysis CKD is associated with increased mortality and progression to end-stage kidney disease [10,11,12,31].

In severe sarcopenia, age was the dominant independent determinant, while the sarcopenic index again failed to provide any clinically meaningful information. Even in patients with reduced kidney function, the index did not retain independent significance. This challenges the assumption that creatinine- and cystatin C–based indices are especially suitable in kidney diseases populations, where metabolic disturbances, inflammation, and uremic toxicity substantially alter biomarker behavior [6,29,32].

### 4.4. Nutritional Status as a Central Determinant

A key contribution of this study is the consistent observation that nutritional proxies appear to outperform composite biochemical indices in identifying clinically relevant sarcopenia. Lower BMI emerged as the most robust independent predictor of confirmed sarcopenia across all analyses. This finding aligns with epidemiological and mechanistic data linking low BMI to chronic energy deficit, inflammation, anabolic resistance, and impaired muscle protein synthesis—core mechanisms in sarcopenia pathophysiology [2,33].

In kidney failure, these mechanisms are further exacerbated by anorexia, dietary protein restriction, metabolic acidosis, and mineral metabolism disorders [6,34,35]. Although BMI does not distinguish lean from fat mass, its consistent association with sarcopenia severity suggests that long-term nutritional status may be more informative than short-term biochemical surrogates.

Serum albumin showed modest univariable associations but did not retain independent significance, consistent with its interpretation as a marker of inflammation rather than muscle mass [36].

### 4.5. Clinical and Research Implications

From a clinical perspective, these findings argue against the routine use of the sarcopenic index for sarcopenia screening or diagnosis in older adults, particularly in patients with kidney insufficiency. Instead, they support guideline-based approaches that prioritize functional assessment and simple anthropometric evaluation [1,5].

Concerns regarding low-protein diets, malnutrition, and catabolic consequences remain clinically relevant, particularly in vulnerable older CKD adults [31,37]. Conversely, evidence supports combined lifestyle strategies, including resistance exercise and targeted nutritional interventions, to improve sarcopenia-related outcomes in CKD [38,39,40,41]. From a nutritional and interventional perspective, early identification of low BMI provides a pragmatic entry point for targeted interventions, including dietary optimization and resistance exercise, which have demonstrated benefits in CKD-associated sarcopenia. These intervention-focused data reinforce the clinical importance of identifying sarcopenia early, especially in geriatric patients with kidney function impairment, where risk and potential benefit of intervention are substantial.

From a research standpoint, our results highlight the need for biomarkers and assessment strategies that capture muscle quality, neuromuscular integrity, and longitudinal change, rather than static biochemical ratios. Imaging-based and integrative approaches appear more promising in CKD-related sarcopenia [18,22].

Future studies specifically designed to assess performance- and decision-based metrics would be required to formally address this question.

By demonstrating the lack of an independent association between the sarcopenia index and EWGSOP2-defined sarcopenia phenotypes, the present findings help delineate the appropriate scope of creatinine–cystatin C–based indices in sarcopenia research. This clarification is particularly relevant in populations with reduced kidney function, where biochemical markers may be disproportionately influenced by non-muscle determinants and risk being misapplied as surrogate indicators of muscle status.

Several limitations merit consideration. First, the high prevalence of probable sarcopenia reflects the geriatric clinical setting and may limit generalizability to community-based populations. Second, hydration status may influence BIA-derived muscle mass estimates, particularly in individuals with kidney dysfunction, potentially attenuating associations with biochemical indices. Importantly, the study was not designed to formally assess diagnostic performance or clinical decision-making utility, which would require discrimination, calibration, interaction testing, and decision-analytic methods. Accordingly, the present findings should be interpreted as associative rather than as an assessment of clinical usefulness. Finally, although the sarcopenic index is conceptually attractive, its underlying assumptions are substantially challenged in CKD, where inflammation and metabolic disturbances affect both creatinine and cystatin C.

These findings are particularly relevant in geriatric nephrology, where pragmatic, low-burden assessments are essential.

## 5. Conclusions

In conclusion, a creatinine–cystatin C–based sarcopenic index did not demonstrate a meaningful association with EWGSOP2-defined probable sarcopenia, the most uniformly assessable EWGSOP2 stage, in older adults, including those with reduced kidney function. Exploratory analyses in more advanced sarcopenia stages did not provide additional associative information. These findings support the continued prioritization of functional assessment and simple anthropometric measures within guideline-based sarcopenia evaluation frameworks.

## Figures and Tables

**Table 1:** Prevalence of sarcopenia and its components according to EWGSOP2 criteria.

Criterion	With, *n* (%)	Without, *n* (%)
**Probable sarcopenia (*n* = 396)**	347 (87.6)	49 (12.4)
HGS < 27 kg (men), <16 kg (women) (*n* = 396)	262 (66.1)	153 (33.9)
Chair stand test > 15 s (*n* = 314)	222 (70.7)	92 (29.3)
**Confirmed sarcopenia (*n* = 218)**	95 (43.6)	123 (56.4)
Low ASM (*n* = 142)	12 (8.5)	130 (91.5)
Low ASMI (*n* = 142)	48 (33.8)	94 (66.2)
CT-derived low muscle mass (*n* = 157)	86 (54.8)	71 (45.2)
**Severe sarcopenia (*n* = 178)**	25 (14)	153 (86)
Low gait speed ≤ 0.8 m/s (*n* = 284)	251 (88.4)	33 (11.6)
SPPB score ≤ 8 (*n* = 396)	340 (85.8)	56 (14.2)
TUG ≥ 20 s (*n* = 374)	202 (54.0)	172 (46.0)

EWGSOP2, European Working Group on Sarcopenia in Older People 2; HGS, handgrip strength; ASM, appendicular skeletal muscle mass; ASMI, appendicular skeletal muscle mass index; CT, computed tomography; SPPB, Short Physical Performance Battery; TUG, Timed Up and Go test. Sample size (*n*) varies according to data availability for each sarcopenia component. Probable sarcopenia was assessed in the entire cohort, whereas confirmed and severe sarcopenia were evaluated in subsets with available muscle mass and physical performance data.

**Table 2:** Correlations between sarcopenic index and sarcopenia-related parameters.

Parameter	*n*	Total r (*p*)	Women r (*p*)	Men r (*p*)
HGS max	396	0.21 (**<0.001**)	0.15 (**0.010**)	0.14 (0.176)
Chair stand test	314	−0.17 (**0.002**)	−0.18 (**0.007**)	−0.13 (0.242)
ASM	142	0.11 (0.199)	−0.16 (0.126)	0.26 (0.127)
ASMI	142	0 (0.985)	−0.20 (**0.048**)	0.10 (0.575)
CT muscle mass	157	0.15 (0.086)	0.08 (0.437)	0.06 (0.741)
SPPB score	396	0.21 (**<0.001**)	0.25 (**<0.001**)	0.15 (0.126)
TUG	374	−0.13 (**0.019**)	−0.16 (**0.010**)	−0.12 (0.242)
Gait speed	284	0.11 (0.078)	0.06 (0.365)	0.10 (0.428)

Abbreviations: HGS, handgrip strength; ASM, appendicular skeletal muscle mass; ASMI, appendicular skeletal muscle mass index; CT, computed tomography; SPPB, Short Physical Performance Battery; TUG, Timed Up and Go test. Correlations were assessed using Spearman’s rank test. Sample size (*n*) reflects the number of participants with available data for each parameter. Values in bold denote statistical significance.

**Table 3:** Clinical and laboratory characteristics according to probable sarcopenia status.

Variable	No Probable Sarcopenia (*n* = 49)	Probable Sarcopenia (*n* = 347)	*p*-Value
Age (years)	76.51 ± 7.29	82.84 ± 6.99	<0.001
BMI (kg/m^2^)	31.49 ± 6.08	28.23 ± 7.08	0.002
Hemoglobin (g/dL)	13.5 (12.3–14)	12.7 (11.3–13.85)	0.008
Albumin (g/dL)	4.4 (4.3–4.6)	4.2 (3.9–4.4)	<0.001
Vitamin D (ng/mL)	27.86 (19.14–34.53)	21.84 (13.36–33.58)	0.047
CRP (mg/L)	0.33 (0.16–0.81)	0.42 (0.16–1.54)	<0.001
eGFR (Cr, mL/min/1.73 m^2^)	67.93 (55.32–86.56)	63.51 (43.56–84.52)	0.038
Cystatin C (mg/L)	1.3 (1.2–1.7)	1.5 (1.2–1.95)	0.007

Abbreviations: BMI, body mass index; CRP, C-reactive protein; eGFR, estimated glomerular filtration rate. Data are presented as mean ± SD or median (IQR), as appropriate. Group comparisons were performed using Student’s *t*-test or Mann–Whitney *U* test, according to data distribution. Sample size reflects participants with available data for each variable.

**Table 4:** Clinical and laboratory characteristics according to probable sarcopenia status in patients with eGFR < 60 mL/min/1.73 m^2^.

Variable	No Probable Sarcopenia (*n* = 16)	Probable Sarcopenia (*n* = 160)	*p*-Value
Male sex, *n* (%)	6 (37.5)	34 (21.3)	0.206
Age (years)	79.83 ± 5.77	84.90 ± 6.34	**0.002**
BMI (kg/m^2^)	29.74 ± 6.06	28.18 ± 6.84	0.381
Hemoglobin (g/dL)	12.8 (11.55–13.8)	12.1 (11.0–13.3)	0.187
Albumin (g/dL)	4.45 (4.3–4.6)	4.1 (3.8–4.4)	**0.006**
Vitamin D (ng/mL)	26.78 (17.33–33.77)	23.46 (14.22–34.76)	0.376
CRP (mg/L)	0.25 (0.14–0.95)	0.60 (0.21–2.15)	0.151
Uric acid (mg/dL)	6.9 (6.25–7.5)	7.3 (6.3–8.55)	0.343
UACR (mg/g)	13.86 (9.29–34.84)	29.41 (13.09–103.85)	**0.046**
Serum creatinine (mg/dL)	1.24 (1.18–1.44)	1.33 (1.11–1.59)	0.436
eGFR (creatinine-based), mL/min/1.73 m^2^	46.62 (42.44–54.40)	42.18 (33.68–51.56)	0.067
Cystatin C (mg/L)	1.75 (1.4–2.1)	1.95 (1.6–2.4)	0.071
eGFR (CysCr-based), mL/min/1.73 m^2^	41.19 ± 11.99	34.41 ± 10.47	**0.016**
Sarcopenic index	74.6 (63.5–85.19)	70.29 (59.1–82.99)	0.248

Abbreviations: BMI, body mass index; CRP, C-reactive protein; eGFR, estimated glomerular filtration rate; UACR, urinary albumin-to creatinine ratio, cys, cystatin, cr, creatinine. Reduced kidney function was defined based on a single eGFR < 60 mL/min/1.73 m^2^ measurement. Data is presented as mean ± SD or median (IQR). Group comparisons were performed using appropriate parametric or non-parametric tests. Values in bold denote statistical significance.

**Table 5:** Clinical and laboratory characteristics according to confirmed sarcopenia status.

Variable	No Confirmed Sarcopenia (*n* = 123)	Confirmed Sarcopenia (*n* = 95)	*p*-Value
Male sex, *n* (%)	29 (23.6)	27 (28.4)	0.417
Age (years)	79.03 ± 7.1	82.83 ± 6.33	**<0.001**
BMI (kg/m^2^)	30.87 ± 6.17	26.02 ± 6.60	**<0.001**
Hemoglobin (g/dL)	13.2 (11.859–14.15)	12.7 (11.8–13.8)	0.058
Albumin (g/dL)	4.4 (4.1–4.6)	4.2 (3.95–4.4)	**0.001**
Vitamin D (ng/mL)	24.95 (16.62–35.01)	27.14 (14.32–38.03)	0.774
CRP (mg/L)	0.34 (0.14–0.95)	0.28 (0.13–1.58)	0.426
Uric acid (mg/dL)	5.75 (4.65–7.1)	6.1 (5.0–7.55)	0.112
UACR (mg/g)	13.12 (9.39–45.23)	22.04 (11.46–67.65)	0.785
Serum creatinine (mg/dL)	0.93 (0.76–1.16)	0.98 (0.81–1.36)	0.096
eGFR (Cr), mL/min/1.73 m^2^	66.64 (52.67–85.65)	64.16 (44.60–78.72)	0.062
Cystatin C (mg/L)	1.3 (1.1–1.7)	1.5 (1.2–1.8)	**0.038**
eGFR (CysCr), mL/min/1.73 m^2^	57.85 ± 19.8	52.26 ± 19.80	**0.040**
Sarcopenic index	67.69 (60–78.87)	68.18 (58.98–77.32)	0.755

Abbreviations: BMI, body mass index; CRP, C-reactive protein; eGFR, estimated glomerular filtration rate; UACR, urinary albumin over creatinine ratio. Values in bold denote statistical significance.

**Table 6:** Characteristics according to confirmed sarcopenia status in patients with eGFR < 60 mL/min/1.73 m^2^.

Variable	No Confirmed Sarcopenia (*n* = 46)	Confirmed Sarcopenia (*n* = 44)	*p*-Value
Age (years)	82.15 ± 5.95	85.01 ± 5.50	0.020
BMI (kg/m^2^)	29.9 ± 6.19	25.36 ± 5.66	<0.001
Uric acid (mg/dL)	7 (5.7–7.8)	7.45 (6.5–8.5)	0.029

Abbreviations: BMI, body mass index.

**Table 7:** Clinical and laboratory characteristics according to severe sarcopenia status.

Variable	No Severe Sarcopenia (*n* = 153)	Severe Sarcopenia (*n* = 25)	*p*-Value
Age (years)	78.85 ± 6.74	85.27 ± 4.29	<0.001
BMI (kg/m^2^)	29.74 ± 6.62	25.22 ± 5.63	0.001
Albumin (g/dL)	4.4 (4.1–4.6)	4.1 (4.0–4.5)	0.018
eGFR (Cr), mL/min/1.73 m^2^	67.65 (53.48–86.33)	56.62 (45.95–74.74)	0.039
Cystatin C (mg/L)	1.3 (1.1–1.7)	1.6 (1.3–1.8)	0.037
eGFR (CysCr), mL/min/1.73 m^2^	58.61 ± 19.16	49.88 ± 19.63	0.037
Sarcopenic index	67.62 (60.48–78.46)	64.29 (56.40–75.83)	0.242

Abbreviations: BMI, body mass index; eGFR, estimated glomerular filtration rate.

**Table 8:** Characteristics according to severe sarcopenia status in patients with eGFR < 60 mL/min/1.73 m^2^.

Variable	No Severe Sarcopenia (*n* = 54)	Severe Sarcopenia (*n* = 14)	*p*-Value
Age (years)	81.43 ± 4.99	85.22 ± 4.75	0.013
Sarcopenic index	74.64 (64.71–87.14)	62.19 (55–79.33)	0.054

## Data Availability

The research data supporting this study’s findings are not publicly available. Further inquiries can be directed at the corresponding author. The dataset used during the current study is available from the corresponding authors upon reasonable request.

## Supplementary Materials

The following supporting information can be downloaded at: https://www.mdpi.com/article/10.3390/jcm15051782/s1, Table S1: Multivariable logistic regression model for probable sarcopenia (n = 396); Table S2: Multivariable logistic regression for probable sarcopenia in participants with reduced kidney function (n = 176); Table S3: Multivariable logistic regression model for confirmed sarcopenia (exploratory; n = 218); Table S4: Multivariable logistic regression model for severe sarcopenia (exploratory; n = 178).
