# Supplementary material for: Revisiting the Sarcopenic Index in Older Adults with Reduced Kidney Function: Association with EWGSOP2-Defined Probable Sarcopenia
Source: J Clin Med. 2026 Feb 26;15(5):1782. doi: 10.3390/jcm15051782 (PMC12986358; doi:10.3390/jcm15051782)
Supplement: Supplementary file 1 [file jcm-15-01782-s001.zip › jcm-4094544-supplementary.pdf]

Supplementary Table S1. Multivariable logistic regression model for probable sarcopenia (n = 396).

Variable	OR	95% CI	p-value	VIF
Age (per year)	1.12	1.05–1.19	0.001	1.21
Sex (male)	0.91	0.44–1.88	0.801	1.09
BMI (kg/m^2)	0.97	0.92–1.02	0.241	1.34
Hemoglobin (g/dL)	0.95	0.78–1.15	0.602	1.28
Albumin (g/dL)	0.74	0.34–1.61	0.451	1.41
Vitamin D (ng/mL)	0.99	0.97–1.01	0.318	1.12
CRP (mg/L)	1.08	0.92–1.27	0.337	1.18
eGFR (Cr-based, per 10 mL/min/1.73 m^2)	0.96	0.83–1.11	0.572	1.36
**Sarcopenic index**	0.99	0.97–1.02	0.604	1.47

Abbreviations: OR, odds ratio; CI, confidence interval; BMI, body mass index; CRP, C-reactive protein; eGFR, estimated glomerular filtration rate; VIF, variance inflation factor. *All variables were entered simultaneously (Enter method). VIF <2 indicates no relevant multicollinearity.*

Supplementary Table S2. Multivariable logistic regression for probable sarcopenia in participants with reduced kidney function (n = 176).

Variable	OR	95% CI	p-value	VIF
Age (per year)	1.10	1.01–1.21	0.037	1.18
Sex (male)	0.83	0.32–2.16	0.701	1.06
BMI (kg/m^2)	0.98	0.92–1.05	0.588	1.29
Albumin (g/dL)	0.69	0.28–1.70	0.417	1.33
UACR (mg/g)	1.01	0.99–1.02	0.241	1.22
eGFR (Cr–CysC-based, per 10 mL/min/1.73 m^2)	0.89	0.72–1.11	0.302	1.41
**Sarcopenic index**	0.98	0.95–1.02	0.382	1.46

Abbreviations: OR, odds ratio; CI, confidence interval; BMI, body mass index; UACR, urinary albumin-to-creatinine ratio; eGFR, estimated glomerular filtration rate; VIF, variance inflation factor.

Supplementary Table S3. Multivariable logistic regression model for confirmed sarcopenia (exploratory; n = 218)

Variable	OR	95% CI	p-value
Age (per year)	1.03	0.98–1.08	0.243
BMI (kg/m^2)	**0.91**	**0.86–0.96**	**<0.001**

Variable	OR	95% CI	p-value
Albumin (g/dL)	0.81	0.42–1.55	0.524
eGFR (Cr–CysC-based)	0.99	0.97–1.01	0.218
**Sarcopenic index**	1.00	0.97–1.02	0.755

Abbreviations: OR, odds ratio; CI, confidence interval; BMI, body mass index; eGFR, estimated glomerular filtration rate.
Exploratory analysis performed in participants with available muscle mass data.

Supplementary Table S4. Multivariable logistic regression model for severe sarcopenia (exploratory; n = 178)

Variable	OR	95% CI	p-value
Age (per year)	1.12	1.00–1.25	0.048
BMI (kg/m^2)	0.97	0.90–1.04	0.362
Albumin (g/dL)	0.71	0.29–1.72	0.448
eGFR (Cr–CysC-based)	0.98	0.95–1.01	0.197
**Sarcopenic index**	0.99	0.96–1.02	0.418
